# Case Report: Metagenomic Next-Generation Sequencing in Diagnosis of *Legionella pneumophila* Pneumonia in a Patient After Umbilical Cord Blood Stem Cell Transplantation

**DOI:** 10.3389/fmed.2021.643473

**Published:** 2021-06-11

**Authors:** Yangyan Wang, Yuanyuan Dai, Huaiwei Lu, Wenjiao Chang, Fan Ma, Ziran Wang, Zhican Liu, Xiaoling Ma

**Affiliations:** ^1^Department of Clinical Laboratory, Affiliated Provincial Hospital of Anhui Medical University, Hefei, China; ^2^Department of Clinical Laboratory, First Affiliated Hospital of University of Science and Technology of China, Hefei, China; ^3^Department of Clinical Laboratory, Peking Union Medical College Hospital, Beijing, China; ^4^Nanjing Medical laboratory, Beijing Genomics Institute, Nanjing, China

**Keywords:** metagenomic next-generation sequencing, *Legionella pneumophila*, myelodysplastic syndrome, umbilical cord blood stem cell transplantation, hospital acquired

## Abstract

We report a case of hospital-acquired *Legionella* pneumonia that was detected by metagenomic next-generation sequencing (mNGS) of blood from a 7-year-old girl after umbilical cord blood stem cell transplantation (UCBT) with myelodysplastic syndrome. UCBT is traditionally associated with an increased risk of infection, particularly during the first 3 months after transplantation. Controlling interstitial pneumonia and severe infection is the key to reducing patient mortality from infection. *Legionella pneumophila* can cause a mild cough to rapidly fatal pneumonia. After mNGS confirmed that the pathogen was *L. pneumophila*, azithromycin, cefoperazone sulbactam, and posaconazole were used for treatment, and the patient's temperature decreased and remained normal. The details of this case highlight the benefits of the timely use of metagenomic NGS to identify pathogens for the survival of immunocompromised patients.

## Introduction

*Legionella*, which is widely found in the natural environment, including in water sources and soil, includes 58 species and 3 subspecies (1). More than 90% of legionellosis is caused by *L. pneumophila*, of which Lp1 is the most common strain (1–4). Mortality from *Legionella* depends on the severity of the disease, the suitability of the initial antimicrobial treatment, the location where the *Legionella* infection was contracted, and host factors (2, 5). Immunocompromised patients are the most susceptible hosts. *Legionella* is a commonly misdiagnosed pathogen that causes severe hospital-acquired pneumonia, and multiple studies have shown it to be both underdiagnosed and undertreated (2). But mNGS technology based on high-throughput sequencing has been widely studied for use as a non-targeted and broad-spectrum pathogen screening technology for identifying clinically moderate and severe infections (6, 7). Here, we report the first case of an umbilical-cord blood stem cell patient with pulmonary *L. pneumophila* infection who was diagnosed by mNGS before the blood culture results were obtained.

## Case Description

A 7-year-old girl with refractory anemia with excess blasts and type 2 myelodysplastic syndrome was admitted to the hematology department in March 2019, and blood analysis showed that the number of primordial blood cells had reached 19%. The patient was treated with allogeneic cord blood stem cell transplantation under electrocardiographic monitoring; 15 days after transplantation, the patient's neutrophils exceeded 0.5 × 10^9^/L, and thus she was transferred to the general ward for further treatment.

**Figure 1:**
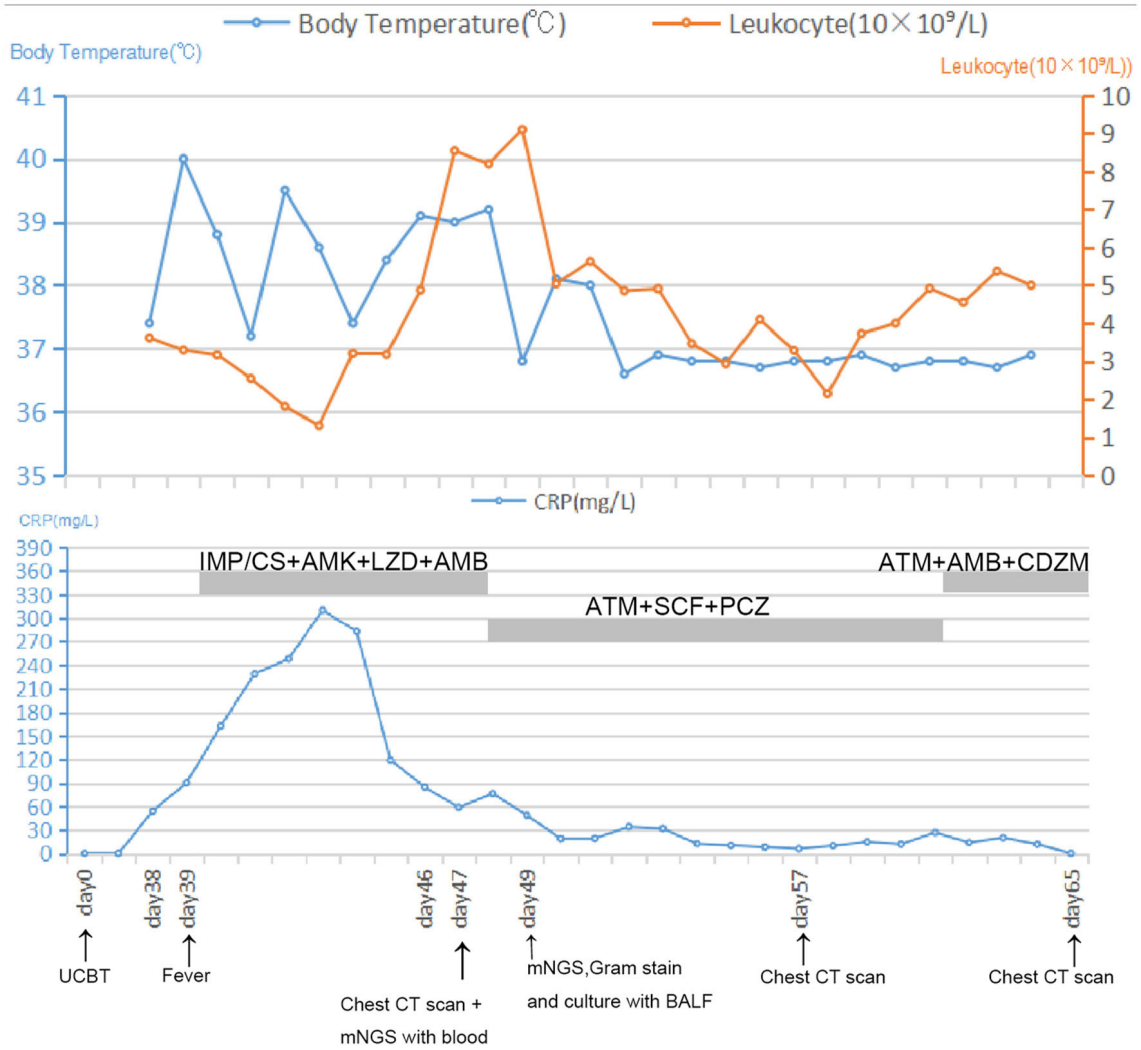
Timeline with relevant data from the episode of care; body temperature curves, leukocyte counts, and CRP. Major events are indicated with arrows. Yellow line shows the leukocyte counts in peripheral blood. Blue line on the top shows body-temperature values. Blue line on the bottom shows CRP values. Horizontal thick gray lines show the medications administered: IMP/CS, Imipenem and cilastatin sodium; AMK, Amikacin; LZD, Linezolid; AMB, Amphotericin B; ATM, Azithromycin; SCF, Sulbactam and cefoperazone sodium; PCZ, Posaconazole; CDZM, Cefodizime.

**Figure 2:**
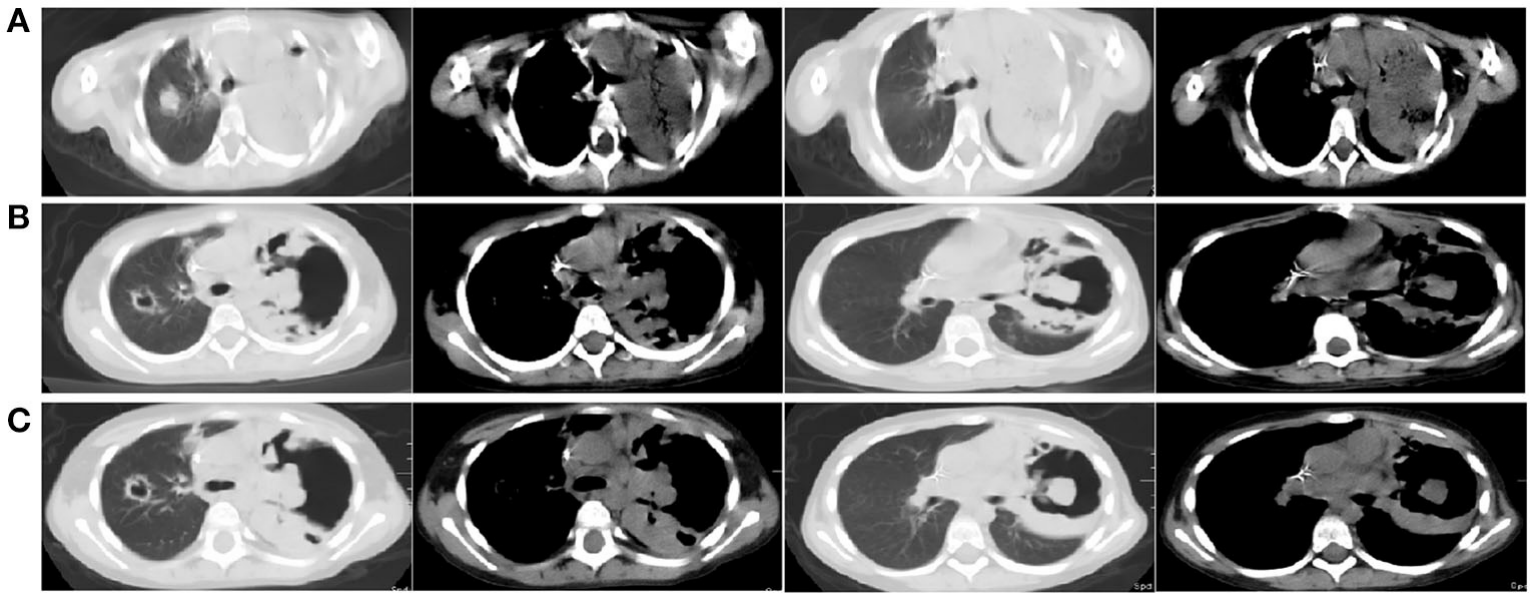
**(A)** Chest CT scan showing double pneumonia: the upper lobe of the left lung was consolidated; **(B)** Chest CT scan showing inflammation in both lungs after 10 days of treatment with azithromycin; **(C)** Chest CT scan revealed that multiple lung lesions were absorbed 18 days later.

**Figure 3:**
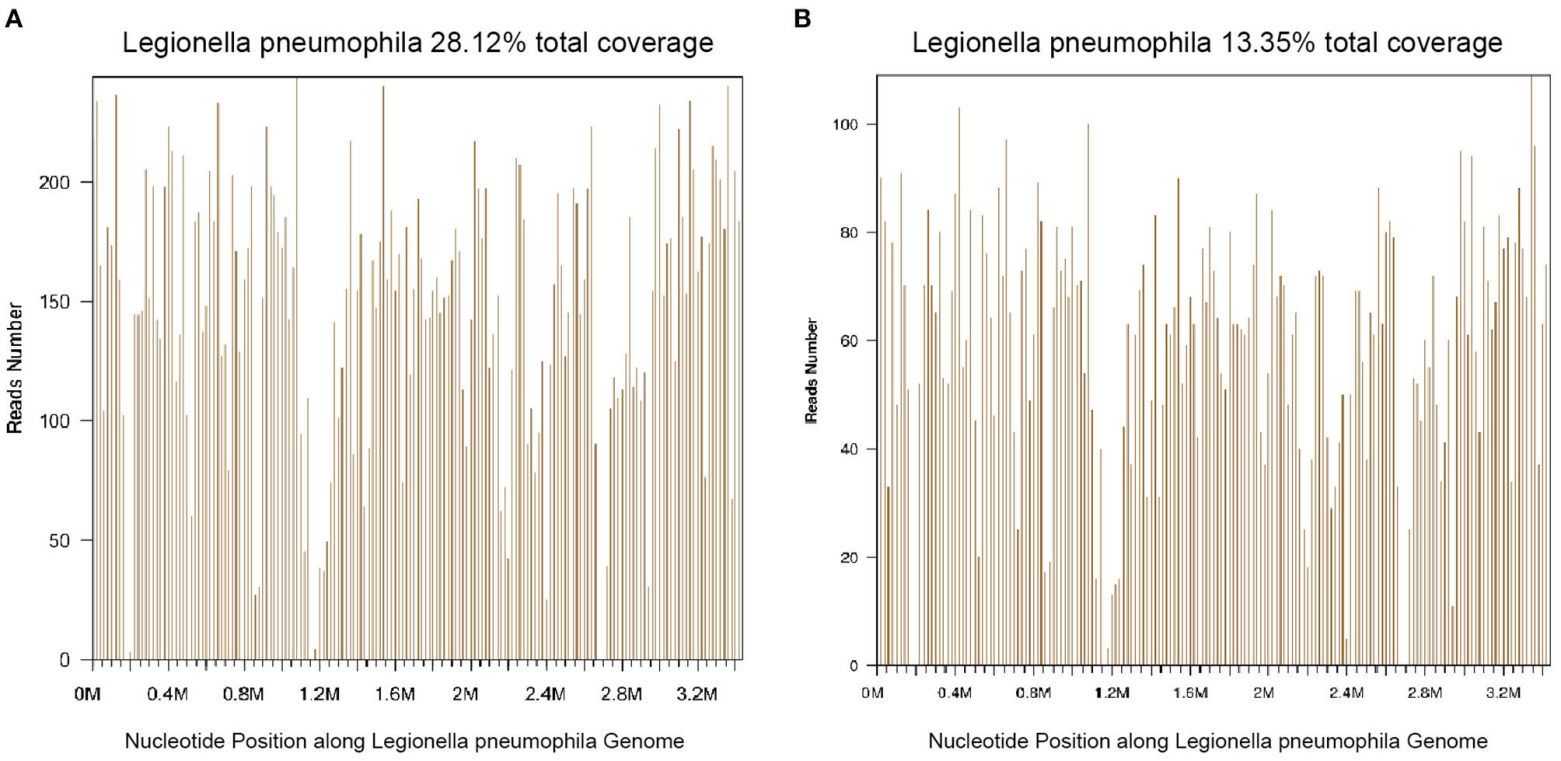
Diagnosis of *Legionella pneumophila* infection using mNGS. **(A)** The majority of reads mapped to the *L. pneumophila* genome with coverage of 28.12%. **(B)** The majority of reads mapped to the *L. pneumophila* genome with coverage of 13.35%.

**Figure 4:**
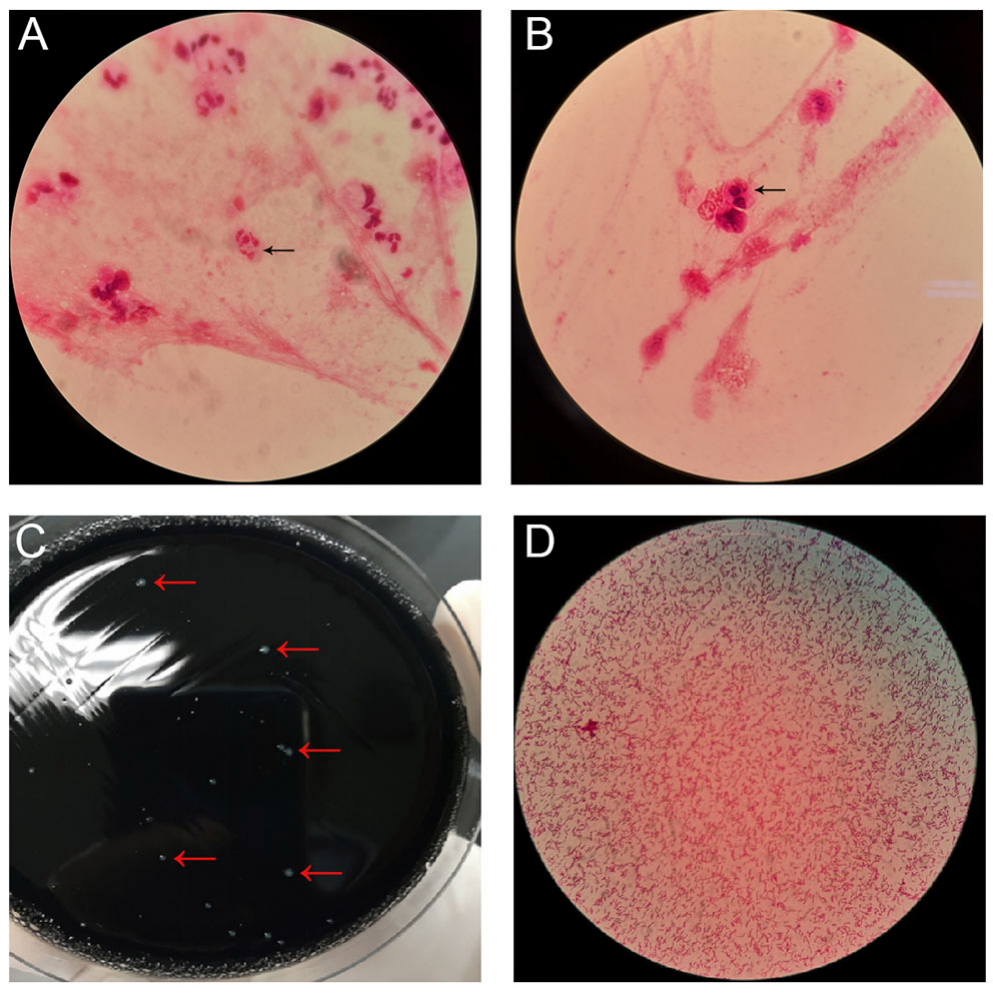
**(A,B)** Gram stain of bronchoalveolar lavage fluid (BALF) with arrows indicating *Legionella pneumophila*, magnification ×1,000. **(C)** *L. pneumophila* colonies on MWY selective agar (red arrows) from BALF. **(D)** Gram stain of *L. pneumophila* colonies (magnification ×1,000).

Thirty-nine days after transplantation, the patient developed a high fever, and her blood neutrophil count and inflammation indicators increased (Figure 1). Therefore, hematologists treated the patient with imipenem, cilastatin, amikacin, linezolid, and liposomal amphotericin B. After 1 week of treatment, the patient's body temperature had not returned to normal, and CT showed double pneumonia, in which the upper lobe of the left lung was consolidated (Figure 2A). We sent the patient's blood samples for mNGS, and 8,112 raw reads belonged to *L. pneumophila* (Figure 3A). The anti-infection regimen was changed to azithromycin combined with cefoperazone, sulbactam, and posaconazole on May 10, 2019 (Figure 1). We then tested the urine for *Legionella* antigen and sent the bronchoalveolar lavage fluid (BALF) for Gram staining, mNGS, and culture on *Legionella* MWY selective agar. The phagocytosis of gram-negative bacilli by leukocytes in BALF was observed under a microscope (Figures 4A,B). The number of raw reads of *L. pneumophila* in BALF was 1,960 (Figure 3B). Six days later, several off-white colonies were visible on the MWY medium (Figure 4C). After Gram staining of the colonies, a large number of gram-negative bacilli were observed under microscopy (Figure 4D). The off-white colonies grown on the MWY medium were subsequently identified as *L. pneumophila* using time-of-flight mass spectrometry. The patient's body temperature returned to normal after 1 day of treatment with azithromycin and remained normal for 1 week (Figure 1), but 10 days after initiating azithromycin treatment, a CT scan still showed inflammation in both lungs (Figure 2B). A CT scan revealed that multiple lung lesions were absorbed 18 days later (Figure 2C). Finally, the patient's general condition improved after follow-up treatment, and she was discharged from hospital on June 3.

## Discussion

The most susceptible hosts of hospital-acquired *Legionella* pneumonia are immunocompromised patients, including organ transplant recipients (8) and cancer patients (9), as well as those receiving glucocorticoid therapy. Multiple studies have shown *Legionella* infections to be underdiagnosed and undertreated (1, 10, 11). In this case report, the patient was fortunate to be treated in time and was discharged from the hospital in good health.

There are many kinds of *Legionella* detection techniques. The immunological methods are limited by the patient's immune status and cross-reactions with other pathogens (12), and at present, this technology can only detect *L. pneumophila* serogroups 1, 3, and 6 (13). For immunocompromised patients, it is important to identify the infectious agent as early as possible in the evolution of the disease. The culture of *Legionella* species from respiratory specimens is the gold standard for the diagnosis of *Legionella* pneumonia (14); however, a *Legionella* culture requires more than 3 days and a medium containing L-cysteine (15, 16). When the patient is in the acute infection phase, samples should be quickly transported to the laboratory for culture, preferably before initiating antimicrobial therapy. The diagnosis of Legionnaires' disease is confirmed by means of culture in only 5% of cases (1). The widely used time-of-flight mass spectrometry detection method can only be used to identify bacterial species after the colonies are cultivated by traditional methods. Thus, the turn-around time is too long for critically ill patients. Obviously, the culture results in this report were obtained very much later than the mNGS results. Other techniques for detecting *Legionella* nucleic acids include PCR (1), isothermal amplification (17), probe hybridization (18), and second-generation sequencing.

This is the first report describing the use of mNGS to detect hospital-acquired *Legionella* pneumonia in a patient after umbilical cord blood transplantation. NGS has unique advantages for the detection of pathogens that are difficult to cultivate (19), especially for cases without target pathogens (20, 21). In addition, mNGS is extremely suitable for detecting unknown pathogens (22, 23), rare pathogens (24, 25), and between-species transmitted pathogens (26, 27). Of course, there are also shortcomings, such as high testing costs, analytical sensitivity, a complex laboratory workflow, and susceptibility to contamination (28). In this case, the mNGS provided directions for choosing traditional diagnosis methods, and the cultured colonies and immunological results were consistent with the mNGS. Although mNGS is relatively expensive, the patient's family considered it worthwhile to identify the pathogen as early as possible to reduce the patient's symptoms. We believe that, with the reductions in sequencing costs and the continuous improvements in medical standards in China, mNGS-based pathogenic diagnosis can be increasingly used to greatly improve the pertinence and timeliness of clinical pathogenic treatment.

## Data Availability Statement

The datasets for this study can be found in the NCBI https://www.ncbi.nlm.nih.gov/bioproject/PRJNA722154 under accession number PRJNA722154.

## Ethics Statement

Written informed consent was obtained from the minor(s)' legal guardian/next of kin for the publication of any potentially identifiable images or data included in this article.

## Author Contributions

YW and YD designed the study. HL, WC, and ZW helped collect data. ZL performed the statistical analysis. FM and XM revised the manuscript, which was written by YW. All authors read and approved the final manuscript.

## Conflict of Interest

The authors declare that the research was conducted in the absence of any commercial or financial relationships that could be construed as a potential conflict of interest.
